# ER stress and unfolded protein response (UPR) signaling modulate GLP-1 receptor signaling in the pancreatic islets

**DOI:** 10.1016/j.mocell.2023.12.002

**Published:** 2023-12-15

**Authors:** Yurong Gao, Hanguk Ryu, Hyejin Lee, Young-Joon Kim, Ji-Hye Lee, Jaemin Lee

**Affiliations:** 1 Department of New Biology, Daegu Gyeongbuk Institute of Science and Technology (DGIST), Daegu 42988, Republic of Korea; 2 Department of Life Sciences, Gwangju Institute of Science and Technology (GIST), Gwangju 61005, Republic of Korea; 3 New Biology Research Center, DGIST, Daegu 42988, Republic of Korea; 4 Well Aging Research Center, DGIST, Daegu 42988, Republic of Korea

**Keywords:** Endoplasmic reticulum stress, G protein-coupled receptor signaling, Glucagon-like peptide-1, Glucagon-like peptide-1 receptor, Type 2 diabetes

## Abstract

Insulin is essential for maintaining normoglycemia and is predominantly secreted in response to glucose stimulation by β-cells. Incretin hormones, such as glucagon-like peptide-1 (GLP-1) and glucose-dependent insulinotropic polypeptide, also stimulate insulin secretion. However, as obesity and type 2 diabetes worsen, glucose-dependent insulinotropic polypeptide loses its insulinotropic efficacy, whereas GLP-1 receptor (GLP-1R) agonists continue to be effective owing to its signaling switch from Gs to Gq. Herein, we demonstrated that endoplasmic reticulum (ER) stress induced a transition from Gs to Gq in GLP-1R signaling in mouse islets. Intriguingly, chemical chaperones known to alleviate ER stress, such as 4-PBA and TUDCA, enforced GLP-1R’s Gq utilization rather than reversing GLP-1R’s signaling switch induced by ER stress or obese and diabetic conditions. In addition, the activation of X-box binding protein 1 (XBP1) or activating transcription factor 6 (ATF6), 2 key ER stress-associated signaling (unfolded protein response) factors, promoted Gs utilization in GLP-1R signaling, whereas Gq employment by ER stress was unaffected by XBP1 or ATF6 activation. Our study revealed that ER stress and its associated signaling events alter GLP-1R’s signaling, which can be used in type 2 diabetes treatment.

## INTRODUCTION

The high incidence of diabetes has occurred globally in recent decades. In 2015, it was estimated that 415 million adults worldwide were affected by diabetes mellitus; by 2040, the number is predicted to rise to 642 million among individuals 20 to 79 years of age (Ogurtsova et al., 2017). Type 2 diabetes (T2D) is more prevalent in adults and accounts for 90% of diabetic patients. In addition to genetic predisposition, an increase in obesity has critically contributed to the global epidemic of T2D (Chen et al., 2011). The T2D is a chronic metabolic disorder characterized by hyperglycemia, in which the body loses proper control of glucose levels in the blood. Diabetic long-term complications include coronary artery disease, limb amputation, diabetic retinopathy, and diabetic nephropathy (Kubota et al., 2017, Mazzone et al., 2008).

Impaired insulin secretion is a hallmark of T2D. It is crucial for glucose homeostasis that pancreatic β-cells properly secret insulin. Glucose-stimulated insulin secretion (GSIS) is the principal mechanism of insulin secretion, which can be boosted by hormones and neurotransmitters. The incretin hormone axis, connecting the absorption of nutrients in the intestine to pancreatic islet hormone release, is crucial to normal glucose tolerance. The glucagon-like peptide-1 (GLP-1) and glucose-dependent insulinotropic polypeptide (GIP), released by enteroendocrine L- and K-cells following nutritional stimulus, are the most important incretin hormones for preventing postprandial hyperglycemia by insulinotropic effect in a glucose-dependent manner (Yabe and Seino, 2011). The GIP and GLP-1 exert their effects by interacting with specific G protein-coupled receptors (GPCRs), the GIP receptor (GIPR) and GLP-1 receptor (GLP-1R), both of which are abundantly expressed in β-cells. The GPCRs activate heterotrimeric G proteins, including Gs and Gq, upon binding to their specific ligands. The GLP-1R and GIPR predominantly couple to the Gs (Baggio and Drucker, 2007). Activated Gs stimulates adenylate cyclase and increases intracellular cyclic adenosine monophosphate (cAMP) levels, which bind and activate protein kinase A (PKA) and the guanine nucleotide exchange protein activated by cAMP-2 (Epac2) (Seino and Shibasaki, 2005). The GLP-1R also couples to the Gq, which potentiates glucose-dependent insulin secretion via phospholipase C (PLC)-diacylglycerol/inositol 1,4,5-trisphosphate (Ahren, 8AD). The GLP-1 and GIP are jointly responsible for the incretin action in pancreatic β-cells in healthy subjects (Vilsboll et al., 2003). In T2D, the insulinotropic effect of incretins, which is responsible for approximately 50% of the insulin secreted after nutrient intake, is progressively lost (Holst and Gromada, 2004, Holst et al., 1997, Preitner et al., 2004). Interestingly, although both receptors couple to Gs to promote intracellular cAMP signaling for insulin secretion, GIP-induced insulin secretion is diminished or absent in T2D individuals, whereas the insulinotropic effect of GLP-1R agonists is still preserved (Holst et al., 2021, Nauck and Meier, 2016). A previous study revealed that a shift in G protein signaling of GLP-1R from Gs to Gq allows GLP-1R agonists to stimulate insulin secretion in β-cells exposed to chronic hyperglycemia and subsequent prolonged membrane depolarization (Oduori et al., 2020). This may explain the preserved insulinotropic efficacy of GLP-1 under T2D, as GLP-1 can activate Gq and Gs, whereas GIP preferentially activates Gs (Hussain et al., 2021, Nichols et al., 2020, Oduori et al., 2020). In the pancreatic β-cells and other tissues, metabolic stresses from obesity and hyperglycemia are known to elicit various cellular stresses, including endoplasmic reticulum (ER) stress (Lee and Ozcan, 2014, Lee and Lee, 2022, Lee et al., 2010, So, 2018). Whether such cellular stresses, particularly ER stress, trigger GLP-1R’s signaling switch to Gq remains unknown.

The ER is the central organelle responsible for proper protein folding, quality control, and secretory capacity of transmembrane and secretory proteins such as insulin. Thus, the ER’s proper operation is essential for cell survival. When the protein load in the ER exceeds its folding capacity, ER stress develops (Kim et al., 2012, Walter and Ron, 2011). Pancreatic β-cells are specialized secretory cells designated for massive insulin synthesis and secretion (Lee and Lee, 2022, Liu et al., 2018). Even under physiological conditions, the burden on the β-cell ER is constitutively high and even higher in insulin-resistant states (Arunagiri et al., 2019, Scheuner et al., 2005). Over the past decades, the growing pieces of experimental data suggest that ER stress contributes to β-cell failure in both type 1 and 2 diabetes (Shrestha et al., 2021, Yong et al., 2021). The primary intracellular network activated by ER stress is the unfolded protein response (UPR), which is mediated by 3 major pathways: inositol-requiring enzyme 1α (IRE1α)-X-box binding protein 1 (XBP1), activating transcription factor 6 (ATF6), and protein kinase RNA-like endoplasmic reticulum kinase (PERK)-activating transcription factor 4 (ATF4) (Gao et al., 2019, Lee and Ozcan, 2014, Lee and Lee, 2022). The UPR restores ER homeostasis and also elicits apoptosis in the event that ER stress cannot be resolved (Lee and Ozcan, 2014). Thus, UPR may perform a homeostatic and protective role as well as cause pathologies in β-cells. C/EBP homologous protein (CHOP), one of the PERK-ATF4 pathway’s targets, has been documented to contribute to β-cell death (Lee and Ozcan, 2014, Lee and Lee, 2022). Additionally, we previously discovered that elevated ATF4 action in β-cells during T2D impaired incretin receptor signaling via phosphodiesterase 4D (PDE4D)-mediated suppression of β-cell cAMP signaling (Lee et al., 2023).

In the investigations presented herein, we found that ER stress caused a GLP-1R signaling change between Gs and Gq in mouse islets. Intriguingly, although chemical chaperones, such as 4-phenylbutyrate (4-PBA) and tauroursodeoxycholic acid (TUDCA), are known to alleviate ER stress, 4-PBA and TUDCA further enforced GLP-1R’s use of Gq rather than reversing ER stress-mediated GLP-1R’s signaling switch. In addition, XBP1 and ATF6, signaling elements of UPR, promoted GLP-1R’s utilization of Gs without reversing ER stress-mediated GLP-1R’s signaling switch.

## MATERIALS AND METHODS

### Mouse Studies

C57BL/6J wild-type (WT) or BKS *db/db* mice (8-12 weeks old) were purchased from Jackson Laboratory. Mice were housed in the animal facilities at Daegu Gyeongbuk Institute of Science and Technology in specific pathogen-free conditions under a 12 hours light (7:00 am to 7:00 pm) to 12 hours dark (7:00 pm to 7:00 am) cycle at an ambient temperature of 20 to 26°C. They were fed with a normal chow diet and water ad libitum. All procedures involving animals were approved by and conducted in accordance with the guidelines of the Daegu Gyeongbuk Institute of Science and Technology Animal Care Center. Age-matched male mice were used throughout the study.

### Mouse Islet Isolation and GSIS

Primary mouse islets were isolated from anesthetized C57BL/6J or BKS *db/db* male mice, as described previously, using a collagenase digestion method (Li et al., 2009). First, we clamped the common bile duct using cotton thread. Next, collagenase P (0.8 mg/ml, C9263-1G, Sigma-Aldrich) was perfused into the pancreas through the ampulla. Collagenase was prepared in 1× Hank’s balanced salt solution (1× HBSS, Thermo Fisher Scientific, Cat no. 14175-095) supplemented with 1 mM CaCl_2_ and 0.3% bovine serum albumin. The enlarged pancreas was extracted and dissociated in a shaking incubator (37°C) at 230 rpm for 10 to 15 minutes. We terminated the digestion by adding 25 ml of 1× HBSS containing 5% FBS and vigorously shaking. Islets were separated by centrifuging at 1,000 rpm for 1 minute at 4°C, resuspended, and washed twice in 20 ml of the same HBSS solution. Finally, we poured the islets into a petri dish and purified them by handpicking them under a dissection microscope (Leica S9E). Primary islets were cultured overnight at 37°C in RPMI1640 media containing 25 mM Hepes (Thermo Fisher Scientific, Cat no. 72400-047) supplemented with 10% fetal bovine serum (FBS), 100 units/ml penicillin and 100 μg/ml streptomycin. All experiments of insulin secretion were performed in pancreatic islets under static incubation. Briefly, islets were washed and preincubated for 1 hour in fresh Krebs-Ringers bicarbonate buffer (BioSolution,125 mM NaCl, 3 mM KCl, 1.2 mM CaCl_2_, 1.2 mM MgSO_4_, 1 mM NaH_2_PO_4_, 10 mM HEPES, 22 mM NaHCO_3_, pH 7.4) supplemented with 3 mM glucose and 0.1% bovine serum albumin at 37°C in 5% CO2. Then, the groups of 5 to 6 islets that were size-matched between groups were transferred to a 24-well plate with Krebs-Ringers bicarbonate buffer containing high glucose (17 mM) and/or other chemicals. After incubation for 1 hour at 37°C, the supernatant was obtained. Secreted insulin was measured using a mouse ultrasensitive insulin ELISA Kit (ALPCO, Cat no. 80-INSMSU-E10).

### Intracellular Insulin Content Measurement

We picked 5 islets for each group after islet isolation (as described above) and assessed islet insulin content using a previously published method (Zhu et al., 2021). After incubation in low glucose (3 mM, 1 hour), we sonicated the collected islets in 200 μl of the insulin content extraction buffer (1.4% HCL in 74% ethanol) for 60 seconds using a Bioruptor (Cosmo Bio Co., Ltd). We further diluted samples (1:300) with insulin ELISA sample diluent provided by the manufacturer and assessed insulin content with the insulin ELISA kit.

### Chemicals

Tunicamycin (item no, 11445), MDL-12330A (item no. 14559), YM-254890 (item no. 29735), sodium 4-phenylbutyrate (4-PBA, item no. 11323), tauroursodeoxycholic acid (TUDCA, item no. 15935), 4μ8c (item no. 22110), and Ceapin-A7 (item no. 36113) were purchased from Cayman Chemical. Exendin-4 (Ex4) was obtained from Merck (E7144), whereas IXA4 was purchased from DC Chemicals (Cat no. DC51012). AA147 was purchased from Tocris Bioscience (CAS No. 393121-74-9).

### Real-time Quantitative Polymerase Chain Reaction (PCR) Analysis

Total RNA was isolated from islets using AccuPrep Universal RNA Extraction Kit (Bioneer Corporation, K-3140) according to the manufacturer’s instructions. Approximately 250 ng of total RNA was reverse transcribed using PrimeScript RT Reagent Kit (TaKaRa, Cat no. RR037A). Real-time PCR was performed with a LightCycler 480 Real-Time PCR machine (Roche) and KAPA SYBR FAST Master Mix (Cat no. KK4611). Primer sequences used are listed in Table 1.

**Table 1:** Primer sequences for RT-qPCR

Genes	Forward	Reverse
XBP1s	GGTCTGCTGAGTCCGCAGCAGG	AGGCTTGGTGTATACATGG
ATF6	GGAGTCGCCTTTTAGTCCGGT	CCGGGGCTCCATAGGTCTGA
Ddit3	CCACCACACCTGAAAGCAGAA	AGGTGAAAGGCAGGGACTCA
Hspa5	TCATCGGACGCACTTGGAA	CAACCACCTTGAATGGCAAGA
Calr	CCTGCCATCTATTTCAAAGAGCA	GCATCTTGGCTTGTCTGCAA
Pde1a	GAAGCAAGCGGGGAGCATAG	AAACAGGAATCTTGAAGCGGTT
Pde3a	TCCCAGTCAGGAACCAGCAT	CAAGTTGCTTACGGCCCTC
Pde11a	AACAGGACCTACGATGAACAGG	TGAGGCAGATTCACCCTCGAT
Pde7b	TGCTAGGAGATGTACGACTAAGG	GGGCCTGCGGTATAATCCC
Pde4d	TGTACCGATCTGACAGCGACT	GCTAGCCAAGACCTGAGCAAA
Adcy1	CGAAACTGCATTGAGGACCG	TCTGCAAACAGGATGCTCACA
Adcy2	ATTAGCACCACGGATGTGCC	TGCTTTTGTGCGTTGATCCC
Adcy3	GACTGCCCTCAACCTGTACG	CCTGTCAGTGCCATTGAGCC
Adcy4	CACCTTGACAGTCCCGTGTC	TTCGACTGCTTCCACTGTTTCT
Adcy5	TGGTGGACCGTGTTCTTCATC	CCACAATGTTGGTGCAGGAG
Adcy6	GCTGCGGAGAATCACTGTCT	TCACACCTGTTACCTCACGC
Adcy7	CATGAGTGAGACTGGACGCCT	GGTGGGAAGAGATGAGGTCAAG
Adcy8	CCGCATCTACATCCATCGCT	AGTAGTAGCAGTCCCCCAGG
Adcy9	CCAGACCTCCCTCTGTGAGA	ATTGATGGGCGGCTTGAAGA

RT-qPCR, real-time quantitative PCR.

### Physiological Analysis

Fasting blood glucose levels were measured through the tail blood using a glucose test strip (GCMS ONE). For the glucose tolerance test, mice were fasted overnight (16 hours) and orally administered with glucose solution (2 g/kg). For the insulin tolerance test, 2 IU/kg of human insulin (Eli Lilly) was intraperitoneally administered to mice fasted for 6 hours.

### Histological Staining of Pancreatic Tissues

The mouse pancreas samples were fixed in formalin, dehydrated, and embedded in an FSC22 frozen section media (Leica). Tissues were cut into 5 µm-thick frozen sections and subjected to an immunofluorescence staining procedure. Mouse insulin primary antibody (Santa Cruz Biotechnology, sc-8033) and rabbit glucagon primary antibody (Abcam, ab92517) were used. Sections were subsequently washed and incubated with Alexa Fluor-conjugated secondary antibodies according to the appropriate species (Invitrogen, A32732 and A32723). Then, sections were stained with DAPI (50 ng/ml) for 5 minutes to stain nuclei and mounted with Fluoromount aqueous mounting medium (Sigma, F4680-25ML).

### Statistical Analysis

The data are expressed as means ± standard error of the mean (SEM) for the specified number of biological replicates. Statistical analyses were performed using GraphPad Prism Ver. 7.02 (GraphPad Software, San Diego, CA). The unpaired t-test with Welch’s correction was used to compare 2 groups, while 1- or 2-way analysis of variance (ANOVA) followed by Holm-Sidak’s tests was used to compare 3 or more groups. *P*-values <.05 were considered statistically significant. Where noted, samples were excluded based on Grubbs outlier testing.

## RESULTS

### ER Stress-Triggered GLP-1R’s Signaling Switch From Gs to Gq in the Mouse Islet

Based on a previous report documenting GLP-1R’s Gs to Gq signaling switch under hyperglycemic conditions, we hypothesized that ER stress under hyperglycemia or other metabolic stress under T2D might elicit such a signaling shift in β-cells. We tested this by administering a low dose of tunicamycin (Tm) (asparagine (N)-linked glycosylation inhibitor, 1 μg/ml) for 8 hours to induce an acute ER stress condition in isolated islets. The 8-hour Tm treatment resulted in a significant increase in the expression of genes associated with ER stress (Supplementary Fig. S1A).

**Fig. 1:**
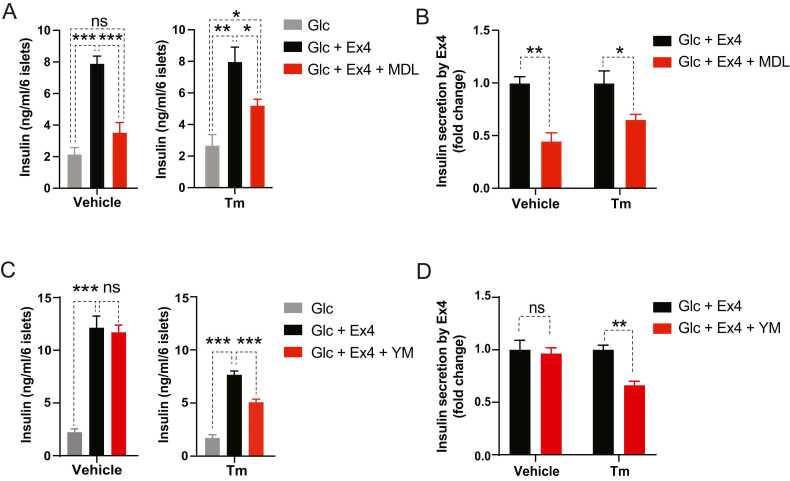
Acute ER stress triggers GLP-1R signaling use of Gq. (A-D) Isolated mouse islets were pretreated with tunicamycin (Tm, 1 μg/ml, 8 hours) and further administered with MDL-12330A (MDL, adenylyl cyclase inhibitor, 10 μM) (A, B) or YM-254890 (YM, Gq inhibitor, 200 nM) (C, D) along with exendin-4 (Ex4, 50 nM) and high glucose (Glc, 17 mM) for an additional 1 hour. (A, C) Insulin secretion and (B, D) its fold change. Data are presented as means ± SEM (n = 4). Statistical analyses were performed using either 1-way ANOVA followed by Holm-Sidak’s test (A, C) or unpaired t-tests with Welch’s correction (B, D). ns, non-significance. *P < .05, **P < .01, ***P < .001.

GLP-1R agonists promote GSIS primarily via a mechanism involving Gs. Accordingly, when islets were treated with Ex4 (50 nM), a GLP-1R agonist, with glucose stimulation (17 mM) for 1 hour in the presence of MDL-12330A (MDL, 10 μM, adenylyl cyclase inhibitor) or YM-254890 (YM, 200 nM, Gq inhibitor) without Tm pretreatment (dimethylsulfoxide), we observed that only Gs signaling inhibition by MDL-12330A, not Gq inhibition (YM-254890), markedly suppressed Ex4-induced insulin secretion (Fig. 1). However, under an acute ER stress condition induced by Tm (Tm), inhibition of Gq (YM-254890) or Gs (MDL-12330A) signaling suppressed Ex4-stimulated insulin secretion, indicating that ER stress triggers GLP-1R’s signaling utilization to Gq (Fig. 1). Our experimental treatment with Tm, Ex4, and Gs and Gq inhibitors did not alter islet’s insulin content (Supplementary Fig. S1B).

### Chemical Chaperones, 4-PBA, and TUDCA, Increased the Incretin Effect by Further Promoting GLP-1R’s Signaling Switch From Gs to Gq

**Fig. 2:**
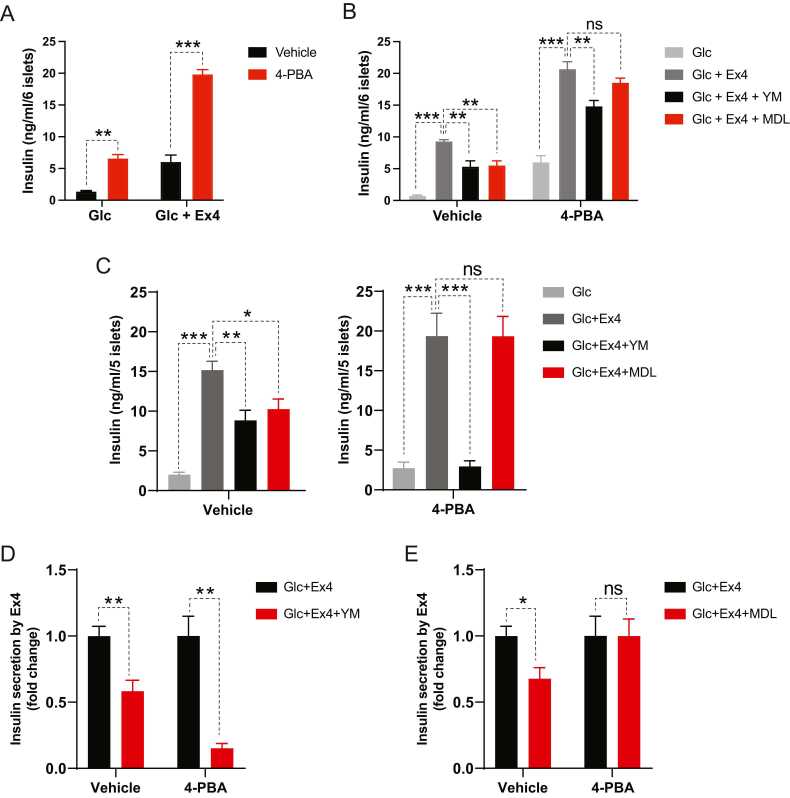
4-PBA, a chemical chaperone, promotes GLP-1R agonist-induced insulin secretion and GLP-1R’s Gq utilization in ER stress-experiencing and *db/db* islets. (A, B) Islet insulin secretion. Isolated lean mouse islets were sequentially treated with 4-PBA (2.5 mM, 24 hours), tunicamycin (1 μg/ml, 8 hours), and high glucose (Glc, 17 mM, 1 hour) with or without Ex4 (50 nM), YM (200 nM), and MDL (10 μM). (C-E) Islets were isolated from 12-week-old *db/db* mice and then pretreated with 4-PBA (2.5 mM, 24 hours) before high glucose with indicated reagents. (C) Insulin secretion and (D, E) its fold change. Data are presented as means ± SEM (n = 4). Statistical analyses were performed using either unpaired t-tests (A, D, E) or 1-way ANOVA (B, C). ns, non-significance. *P < .05, **P < .01, ***P < .001.

Next, we investigated whether relieving ER stress reverses GLP-1R’s signaling switch triggered by ER stress. Earlier studies have shown that treatment with chemical chaperones, such as 4-PBA and TUDCA, alleviates ER stress and increases β-cell survival (Engin et al., 2013, Tang et al., 2012). When mouse islets were pretreated with 4-PBA or TUDCA and then their ER stress was induced with Tm (8 hours), both chemical chaperones significantly facilitated glucose- and Ex4-induced insulin secretion under the acute ER stress condition induced by Tm (Fig. 2A, Supplementary Fig. S2). As shown in Figure 1, inhibition of Gs or Gq signaling with MDL-12330A or YM-254890 in the presence of Tm substantially reduced Ex4-stimulated insulin secretion (Fig. 2B). Intriguingly, while Gq inhibition by YM-254890 significantly decreased Ex4-stimulated insulin secretion in the presence of 4-PBA or TUDCA, Gs inhibition by MDL-12330A had no inhibitory effect on Ex4-stimulated insulin secretion (Fig. 2B, Supplementary Fig. S2B). This suggests that 4-PBA or TUDCA treatment boosts GLP-1R’s signaling usage of Gq rather than reversing the ER stress-induced GLP-1R’s signaling shift to Gs.

In some hyperglycemic mouse models, including KK-Ay obese and diabetic mice, GLP-1R shifted its signaling use from Gs to Gq (Oduori et al., 2020). In hyperglycemic conditions such as obesity and T2D, it has been proposed that pancreatic β-cells experience chronic ER stress. Thus, we examined whether chemical chaperones, such as 4-PBA, modulate GLP-1R’s signaling switch in obese and diabetic *db/db* mouse islets, as we did in our experiments with islets experiencing acute ER stress. In contrast to a 1 g/kg dose of 4-PBA, which was previously reported to improve glucose tolerance and insulin sensitivity (Ozcan et al., 2006), a 100 mg/kg dose of 4-PBA had no effect on body weight, fed blood glucose, or insulin sensitivity (Supplementary Fig. S3A-D). Nevertheless, 4-PBA treatment significantly improved glucose tolerance when glucose was given orally (intragastrically) but did not alter the composition of insulin-producing β-cell and glucagon-producing α-cell within *db/db* islets (Supplementary Fig. S3E-G), indicating that 4-PBA enhanced incretin-stimulated glucose disposal without increasing β-cell numbers. Furthermore, as in our previous experiments with WT islets treated with Tm and 4-PBA (Fig. 2A), 4-PBA administration markedly promoted Ex4-induced insulin secretion in *db/db* islets (Supplementary Fig. S4A and B). Next, we tested whether alleviating ER stress with chemical chaperones also modulates GLP-1R signaling in β-cells of obese and diabetic mice. Similar to our acute ER stress experiments (Figs. 1 and 2B, Supplementary Fig. S2B) and a prior study on other hyperglycemic murine islets (Oduori et al., 2020), Gs and Gq inhibition suppressed the response of *db/db* islets to Ex4 (Fig. 2C-E). When we administered 4-PBA, MDL-12330A lost its inhibitory effect on Gs signaling, whereas Gq inhibition by YM-254890 continued to substantially attenuate Ex4-stimulated insulin secretion (Fig. 2C-E). Collectively, our findings from islets subjected to acute (Tm) and chronic (obesity) ER stress suggest that chemical chaperones facilitate β-cell response to GLP-1R agonists by enhancing GLP-1R’s utilization of Gq rather than Gs. This also suggests that the β-cell’s adaptive response to ER stress is responsible for GLP-1R’s signaling switch under ER stress.

### XBP1 Enhanced GLP-1R’s Gs Utilization Under ER Stress

Among the 3 arms of the UPR, we demonstrated that elevated ATF4 activity in T2D β-cells suppressed the cAMP signaling of incretin receptors through transcriptional activation of cAMP-degrading PDE4D (Lee et al., 2023). Thus, we investigated whether other branches of the UPR pathway (XBP1 and ATF6) are involved in GLP-1R signaling modulation under ER stress conditions.

**Fig. 3:**
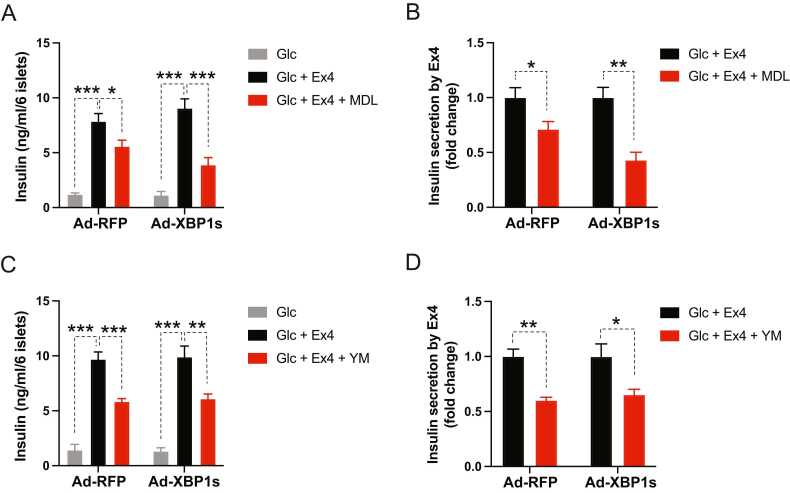
Ectopic expression of active XBP1 (XBP1s) in ER stress-experiencing islets enhances GLP-1R signaling use of Gs. (A-D) Isolated mouse islets were infected with adenovirus expressing red fluorescent protein (Ad-RFP) or XBP1s (Ad-XBP1s) and then pretreated with tunicamycin (8 hours) before introducing indicated reagents for 1 hour. The concentrations of reagents were the same as in previous experiments. (A, C) Islets’ insulin secretion and (B, D) its fold change. Data are presented as means ± SEM (n = 4). Statistical analyses were performed using either 1-way ANOVA (A, C) or unpaired t-tests (B, D). *P < .05, **P < .01, ***P < .001.

First, we ectopically expressed the active form of XBP1 (spliced isoform of XBP1, XBP1s) in the islets using an adenoviral vector, and then we induced ER stress by administering Tm prior to MDL-12330A or YM-254890 treatment. MDL-12330A moderately inhibited Ex4-stimulated insulin secretion in control islets (Ad-RFP) under ER stress, which was further enhanced in the presence of ectopic XBP1s (Fig. 3, Fig. 3). Contrarily, XBP1s expression did not affect the extent of YM-254890-induced suppression of Ex4′s insulinotropic action under ER stress (Fig. 3, Fig. 3).

**Fig. 4:**
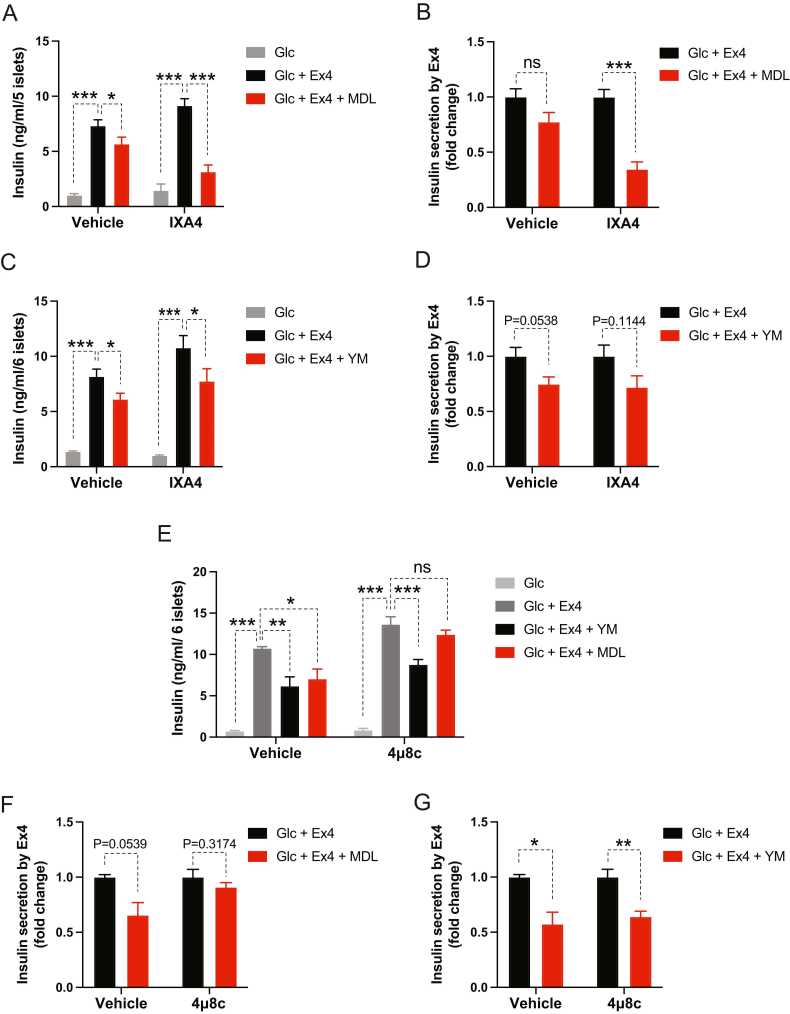
Pharmacological modulation of XBP1s activity leads to altered GLP-1R’s Gs utilization in ER-stress-experiencing islets. (A-G) Isolated mouse islets were pretreated with IRE1α activator (IXA4, 20 μM, 24 hours) (A-D) or inhibitor (4μ8c, 32 μM, 32 hours) (E-G) and then tunicamycin (8 hour) before introducing indicated reagents for 1 hour. The concentrations of reagents were the same as in previous experiments. (A, C, E) Islets’ insulin secretion and (B, D, F, G) its fold change. Data are presented as means ± SEM (n = 4). Statistical analyses were performed using either 1-way ANOVA (A, C, E) or unpaired t-tests (B, D, F, G). ns, non-significance. *P < .05, **P < .01, ***P < .001.

Next, we modulated pharmacologically XBP1s’ activity using an activator (IXA4) or an inhibitor (4μ8c) of XBP1′s activating kinase, IRE1α (Cross et al., 2012, Grandjean et al., 2020, Madhavan et al., 2022). Similar to our ectopic expression of XBP1s, pharmacological activation of IRE1α with IXA4 in islets under ER stress led to further inhibition of Ex4-stimulated insulin secretion by a Gs inhibitor (MDL-12330A) without altering the effect of Gq inhibition with YM-254890 (Fig. 4A-D). Accordingly, when an IRE1α inhibitor, 4μ8c, was added to the islets that had received Tm, insulin secretion stimulated by Ex4 was no longer inhibited by MDL-12330A, and Gq inhibition had no effect on Ex4′s insulinotropism (Fig. 4, Fig. 4, Fig. 4). Genetic or pharmacological modulation of XBP1s’ activity in our study did not change the islet’s insulin content (Supplementary Fig. S1C and D).

### ATF6 Likely Contributed to the Alteration of GLP-1R Signaling by Functioning as XBP1

Next, we investigated the role of ATF6 in GLP-1R signaling. Prior to sequentially introducing Tm, MDL-2330A or YM-254890, and Ex4 with high glucose, we ectopically expressed ATF6 in the islets using an adenoviral vector and observed that ATF6 further enhanced the inhibitory effect of Gs inhibition with MDL-2330A in Ex4-stimulated insulin secretion without any changes from Gq inhibition (YM-254890). This is a similar result to that observed when XBP1 was activated (Supplementary Fig. S5A-C).

Next, we altered ATF6′s activity pharmacologically using an activator (AA147) or an inhibitor (CeapinA7) (Gallagher and Walter, 2016, Paxman et al., 2018, Plate et al., 2016) to determine whether ATF6 modulates GLP-1R’s signaling in the same manner as ectopic ATF6 expression. Similar to ATF6 overexpression, pharmacological activation of ATF6 with AA147 in islets under ER stress led to further inhibition of Ex4-stimulated insulin secretion by Gs inhibition (MDL-12330A) without affecting Gq inhibition on Ex4-stimulated insulin secretion (Supplementary Fig. S6A-C). These outcomes are identical for ATF6 and XBP1 activation. However, unlike XBP1s, when an ATF6 inhibitor, CeapinA7, was added to Tm-treated islets, there was no alteration in Ex4-induced insulin secretion by Gs or Gq inhibition (Supplementary Fig. S6D-F). ATF6 and XBP1 bind to the same promoter and share the majority of their target genes (Lee and Ozcan, 2014, Yamamoto et al., 2004). Based on this, the absence of GLP-1R signaling change with ATF6 inhibition suggests that XBP1 is primarily responsible for GLP-1R’s signaling alteration toward Gs under ER stress in mouse islets, and the observed outcomes of ATF6′s activation are likely due to its shared activation of XBP1 targets. Collectively, our findings suggest that XBP1 and, to a lesser extent, ATF6 direct GLP-1R signaling to utilize Gs without affecting ER stress-triggered GLP-1R’s Gq usage.

### XBP1 and ATF6 Attenuated cAMP-Degrading Phosphodiesterase Expression

In an effort to uncover the probable molecular mechanism of GLP-1R’s signaling switch induced by ER stress and chemical chaperones, we analyzed publicly available transcriptomic data from islets of WT or human islet amyloid polypeptide (hIAPP)-expressing mice, both of which were given either vehicle or 4-PBA (Supplementary Fig. S7) (Montane et al., 2017). hIAPP has been demonstrated to aggregate and cause ER stress in β-cells (Montane et al., 2017). When we examined transcriptional changes of GPCR signaling-related genes, including families of Gα, Gβ, Gγ, adenylate cyclase, PKA, phosphodiesterase (PDE), PLC, and protein kinase C, no marked differences in their expression were observed in WT islets despite 4-PBA administration (Supplementary Fig. S7). Compared to WT islets, hIAPP-expressing islets exhibited alterations in the mRNA expression of some of the G protein γ subunits (Gγ), PKA, PDE, PLC, and protein kinase C families, which were reversed by 4-PBA treatment (Supplementary Fig. S7). However, despite ER stress (hIAPP) and 4-PBA introduction, we were unable to detect notable changes in the expression of GPCR signaling-related genes expected to induce GLP-1R’s signaling switch from Gs to Gq (Supplementary Fig. S7). This suggests that the 4-PBA-induced utilization of GLP-1R signaling to Gq may be due to post-transcriptional changes in GLP-1R signaling.

Previously, we demonstrated that elevated ATF4 action during T2D downregulated incretin receptors’ Gs/cAMP signaling via PDE4D without altering Gq signaling (Lee et al., 2023). In order to determine how XBP1 and ATF6 alter GLP-1R’s signaling change toward Gs, we examined the transcript levels of cAMP-targeting PDEs, which were previously documented to be elevated in their expression in obese and diabetic mice (*db/db*) (Lee et al., 2023), in the islets following ectopic expression of XBP1s or ATF6. Unlike ATF4, XBP1s significantly decreased the transcript levels of *Pde3a*, *Pde4d*, *Pde7b*, and *Pde11a* in the islets (Supplementary Fig. S8A and B). Next, we observed that when ATF6 was expressed ectopically in the islets, *Pde4d* and *Pde11a* expression decreased significantly, similar to XBP1s-expressing islets, while *Pde3a* and *Pde7b* expression did not differ (Supplementary Fig. S8D and E). We did not observe any significant differences in the expression of notable adenylyl cyclases following islet overexpression of XBP1s or ATF6 (Supplementary Fig. S8C and F).

## DISCUSSION

As glucose is an essential source of energy for the body, its metabolism is tightly regulated, particularly by insulin, which is released into the bloodstream by pancreatic β-cells after a meal. Although no panacea has yet arrived to cure T2D, neuronal and hormonal inputs that stimulate insulin secretion have shown promise for adjusting insulin secretion in diabetic individuals. Among these, the incretin hormones GIP and GLP-1 play a central role in regulating insulin secretion. The GIP and GLP-1 use cAMP-dependent intracellular signaling pathways in normal β-cells. However, the ability of GIP to stimulate insulin secretion eventually declines in T2D individuals, and only GLP-1 action remains intact, which explains the therapeutic efficacy of GLP-1R agonists in T2D patients (Nichols et al., 2020).

It was reported that hyperglycemia and persistent depolarization of β-cells led to GLP-1R’s signaling switch from Gs to Gq, whereas GIPR continued to rely on Gs (Oduori et al., 2020). However, its molecular mechanism remains elusive. Previously, we found that ER stress, one of the crucial cellular stresses experienced by pancreatic β-cells under T2D conditions, triggered impaired incretin responses by suppressing cAMP signaling via ATF4-mediated PDE4D expression (Lee et al., 2023). These 2 investigations clarify why GIP loses its insulinotropic efficacy in T2D while GLP-1R agonizts maintain it. Herein, we further demonstrated that ER stress triggered the signaling switch from Gs to Gq in GLP-1R (Fig. 1). Additionally, ER stress-relieving chemical chaperones, such as 4-PBA and TUDCA, promoted GLP-1R agonist (Ex4)-induced insulin secretion in the islets acutely treated with Tm (Fig. 2A, Supplementary Fig. S2A) or from obese and diabetic *db/db* mice (Supplementary Fig. S4). Interestingly, neither 4-PBA nor TUDCA could rectify ER stress-induced GLP-1R’s signaling switch to Gq (Fig. 2, Supplementary Fig. S2). In contrast, they facilitated GLP-1R’s signaling utilization of Gq (Fig. 2, Supplementary Fig. S2), suggesting that GLP-1R’s signaling transition from Gs to Gq under ER stress may be a consequence of the pancreatic β-cell’s response against ER stress.

Our analysis of previously published transcriptomic data from WT or hIAPP-expressing islets receiving 4-PBA did not reveal any noticeable transcriptional changes to explain GLP-1R’s signaling switch to Gq (Supplementary Fig. S7), suggesting that transcriptional changes in GPCR components may not mediate GLP-1R’s signaling change under ER stress and chemical chaperone treatment. Post-translational modulation of GLP-1R and its associated signaling components may modulate GLP-1R’s signaling switch under ER stress, which necessitates further investigation.

In addition, we uncovered that the UPR transcription factors, XBP1 and ATF6, facilitated GLP-1R’s Gs use in β-cells under ER stress in part by downregulating cAMP-targeting PDEs (Fig. 3, Supplementary Figs. S5, S6, and S8), which is the opposite of ATF4, inhibiting incretin receptor’s Gs signaling via elevation of PDE4D under T2D conditions (Lee et al., 2023). In contrast to XBP1, however, pharmacological suppression of ATF6 activity had no discernible effect on GLP-1R signaling under ER stress. This suggests that ATF6′s role in GLP-1R signaling under ER stress may not be as crucial as XBP1s, and GLP-1R’s signaling use to Gs by ATF6 activation may be due to ATF6′s shared activation of XBP1′s targets.

The previous report on GLP-1R’s signaling switch from Gs to Gq (Oduori et al., 2020) and our prior study on ATF4′s role in incretin resistance (Lee et al., 2023) helped us comprehend incretin resistance and sustained GLP-1R agonists' efficacy compared with a loss of GIP’s action under T2D conditions. However, little is known about GLP-1R’s unique signaling switch to Gq other than the fact that it is triggered by hyperglycemia and β-cell depolarization. Here, we found that islet ER stress, which is typically induced during obese and hyperglycemic conditions, initiated GLP-1R’s signaling utilization from Gs to Gq. In addition, chemical chaperones further boosted GLP-1R’s Gq utilization, which is likely part of the β-cell’s response to ER stress to overcome impaired Gs signaling.

It is known that the UPR mediates cellular responses to ER stress (Lee and Ozcan, 2014, Lee and Lee, 2022). Previous studies have documented that XBP1 promotes insulin folding, processing, and secretion (Lee et al., 2011) and helps to maintain β-cell identity (Lee et al., 2022). ATF6 has been reported to contribute to glucose-induced β-cell proliferation (Sharma et al., 2015). However, whether XBP1 and ATF6 play a role in incretin signaling remains unknown. Our findings demonstrated that XBP1 and, to a lesser extent, ATF6 altered GLP-1R’s signaling by facilitating its utilization of Gs under ER stress. ER stress induces UPR activation, including XBP1s and ATF6. However, activated XBP1s and ATF6 were insufficient to increase GLP-1R’s use of Gs signaling under Tm- or obesity-induced ER stress conditions. This implies that Gq utilization under these ER stress conditions outpaced XBP1s- and ATF6-mediated GLP-1R’s Gs usage. Previously, it has been reported that elevated chronic ER stress in the liver of obese mice was accompanied by impaired XBP1s and ATF6 action (Park et al., 2010, Wang et al., 2009). It is also possible that β-cells under obesity and T2D display GLP-1R’s signaling shift to Gq, due in part to insufficient XBP1s’ and ATF6′s action enhancing GLP-1R’s Gs use.

Positively or negatively, the UPR transcription factors XBP1, ATF6, and ATF4 regulate the Gs signaling of the incretin receptor. However, unlike chemical chaperones, they do not contribute to GLP-1R’s signaling switch from Gs to Gq under ER stress. Given that 4-PBA and TUDCA are known to reduce ER stress and its accompanying UPR responses, including XBP1 and ATF6 activation (Lee and Ozcan, 2014), the decreased activity of XBP1 and ATF6 by both chemical chaperones may partly contribute to GLP-1R signaling use of Gq. Nonetheless, the possible involvement of other ER-stress-associated factors in GLP-1R’s signaling transition to Gq needs to be investigated. In conclusion, our study revealed that GLP-1R’s signaling is modulated by ER stress and its associated responses, a finding that has therapeutic implications for the treatment of T2D.

## Author Contributions

Y.G. performed the experiments, and H.R. analyzed the transcriptome data. H.R. and H.L. helped Y.G.’s experiments. Y.G., Y.-J.K., J.-H.L., and J.L. examined the experimental data with contributions from all authors. J.-H.L. and J.L. wrote the manuscript with input from all authors. The study was conceptualized and supervised by Y.G., J.-H.L., and J.L. As the guarantor of this work, J.-H.L. and J.L. had full access to all of the data in the study and took responsibility for their integrity and the accuracy of the data analysis.

## Declaration of Competing Interests

No potential conflicts of interest relevant to this article were reported.
