# Supplementary material for: ER stress and unfolded protein response (UPR) signaling modulate GLP-1 receptor signaling in the pancreatic islets
Source: Mol Cells. 2023 Dec 15;47(1):100004. doi: 10.1016/j.mocell.2023.12.002 (PMC10880082; doi:10.1016/j.mocell.2023.12.002)
Supplement: Supplementary file 1 — Supplementary material [file mmc1.pdf]

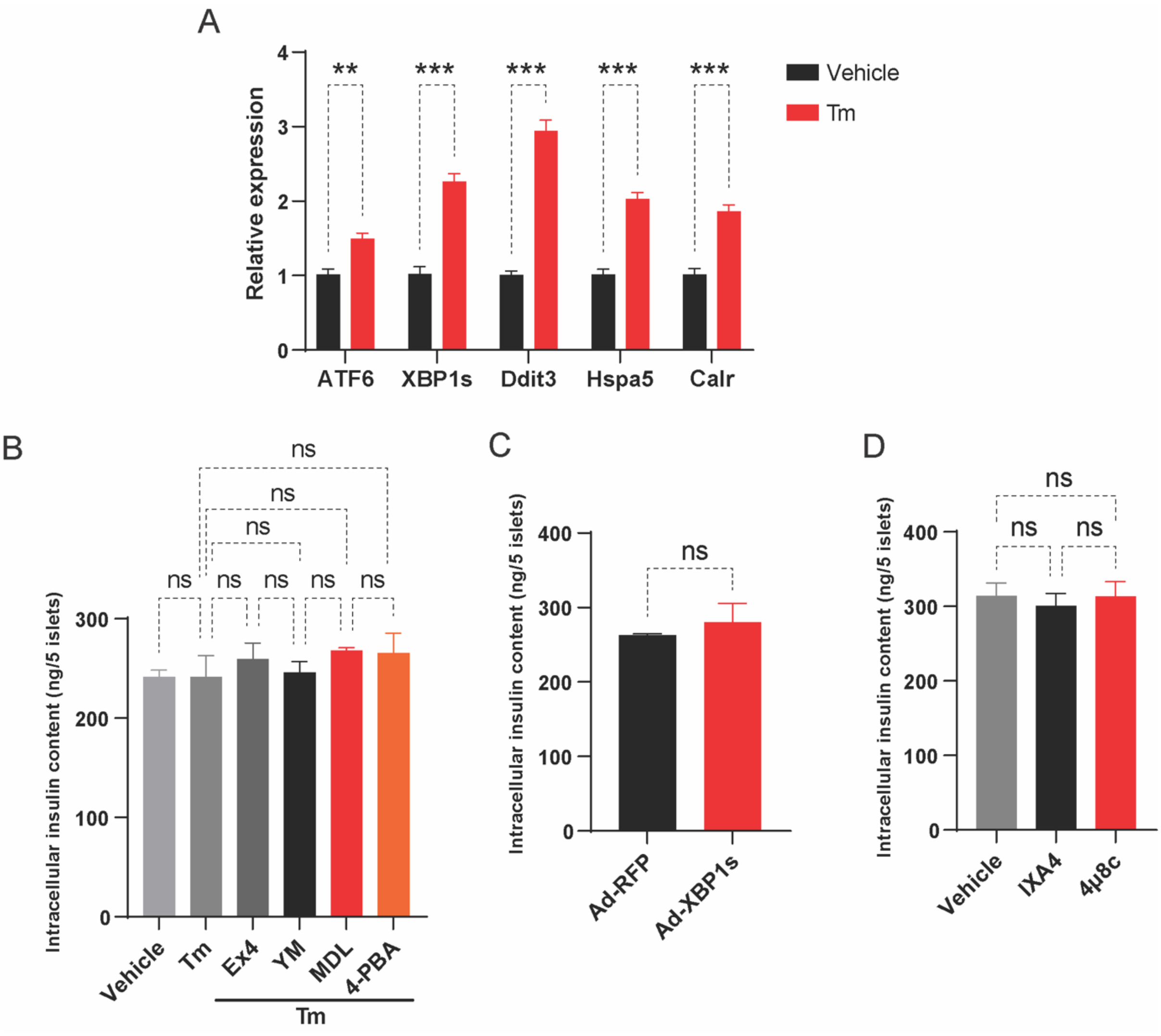

Supplementary Fig. S1. Unchanged insulin content in the islet during the experiments. (A) The mRNA levels of ER stress-associated genes after 8-h tunicamycin treatment (1 μg/ml). (B) Islet insulin content measured by insulin ELISA after tunicamycin (Tm, 1 μg/ml, 8 h), exendin-4 (Ex4, 50 nM, 1 h), YM-254890 (YM, 200 nM, 1 h), MDL-12330A (MDL, 10 μM, 1 h), or 4-PBA (2.5 mM, 24 h). (C–D) Islet insulin content after ectopic expression of XBP1s via adenovirus (C) or pharmacological modulation of XBP1s activity (IXA4, 20 μM, 24 h; 4μ8c, 32 μM, 32 h)(D). Data are presented as means ± S.E.M (n = 4). Statistical analyses were performed by either unpaired t-tests (A,C) or one-way ANOVA (B,D). ** $p < 0.01$, *** $p < 0.001$.

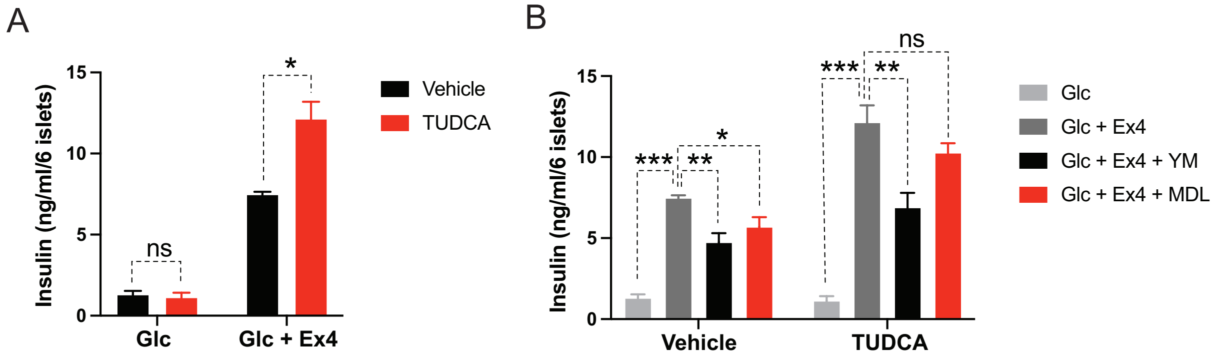

Supplementary Fig. S2. TUDCA promotes GLP-1R agonist-induced insulin secretion and GLP-1R's Gq utilization in islets with ER stress. (A,B) Islet insulin secretion. Isolated islets were sequentially treated with TUDCA (500 μM, 24 h), tunicamycin (1 μg/ml, 8 h), and high glucose (17 mM, 1h) with or without Ex4 (50 nM), YM (200 nM), and MDL (10 μM). Data are presented as means ± S.E.M (n = 4). Statistical analyses were performed by either unpaired t-tests (A) or one-way ANOVA (B). * $p < 0.05$, ** $p < 0.01$, *** $p < 0.001$.

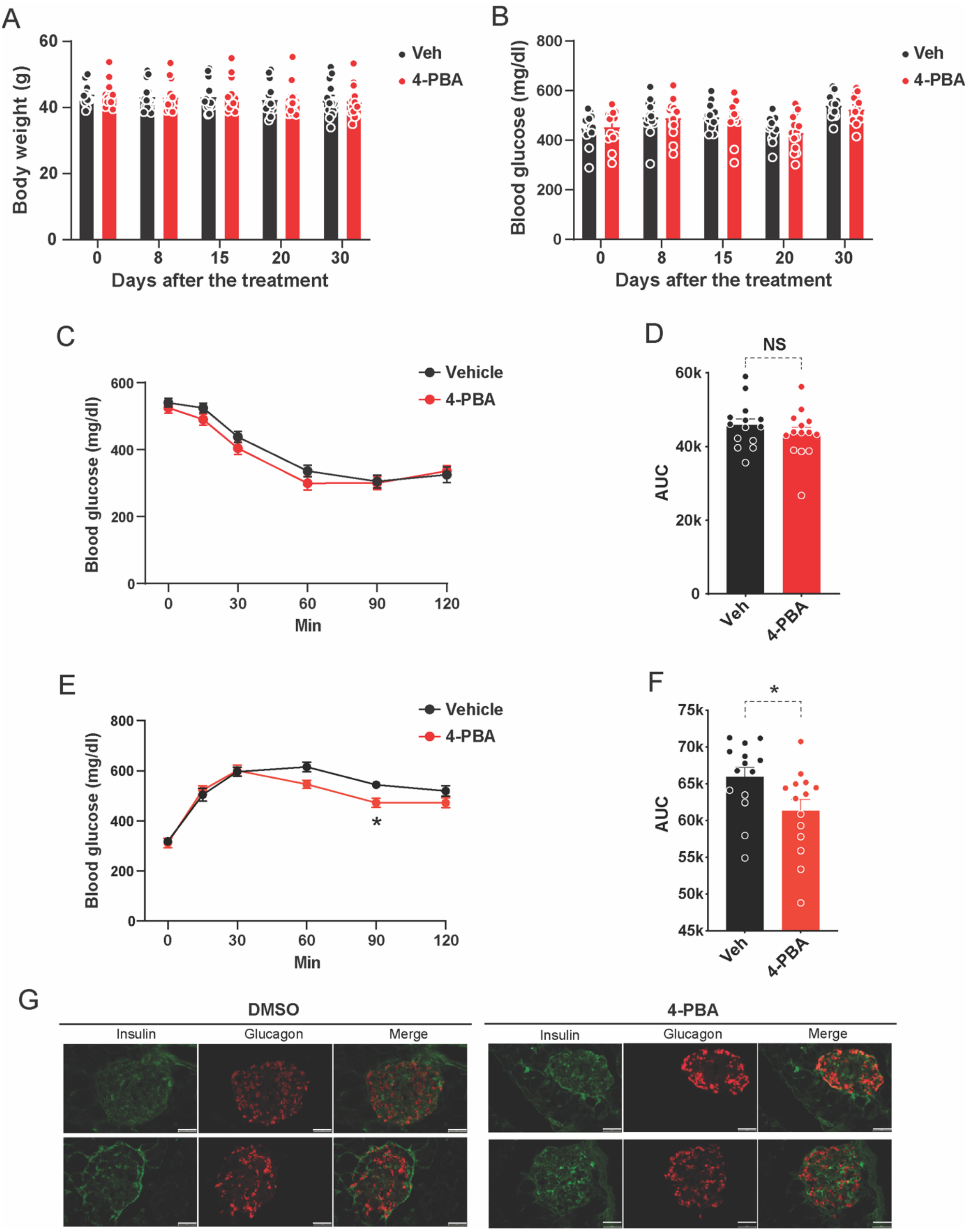
A
Body weight (g)
Days after the treatment
Veh
4-PBA
B
Blood glucose (mg/dl)
Days after the treatment
Veh
4-PBA
C
Blood glucose (mg/dl)
Min
Vehicle
4-PBA
D
NS
AUC
Veh
4-PBA
E
Blood glucose (mg/dl)
Min
Vehicle
4-PBA
*
F
*
AUC
Veh
4-PBA
G
DMSO
4-PBA
Insulin
Glucagon
Merge

Supplementary Fig. S3. 4-PBA promotes incretin-induced insulin secretion in obese and diabetic *db/db* mice. (A–G) 15-wk-old male *db/db* mice intraperitoneally received 4-PBA (100 mg/kg) daily. (A) Body weight and (B) blood glucose levels. Injection started on 15 weeks old *db/db* male mice. (C) Insulin tolerant test and (D) its area under the curve (AUC). (E) Oral glucose tolerant test and (F) its area under the curve (AUC). (G) Representative images of pancreatic islets with insulin- and glucagon-positive cells. The scale bar is 50 μm. Data are represented as mean + S.E.M (n = 14 per group). Statistical analyses were performed by unpaired t tests with Welch's correction (A,B,D,F) or two-way ANOVA followed by Sidak's test (C,E). * $p < 0.05$.

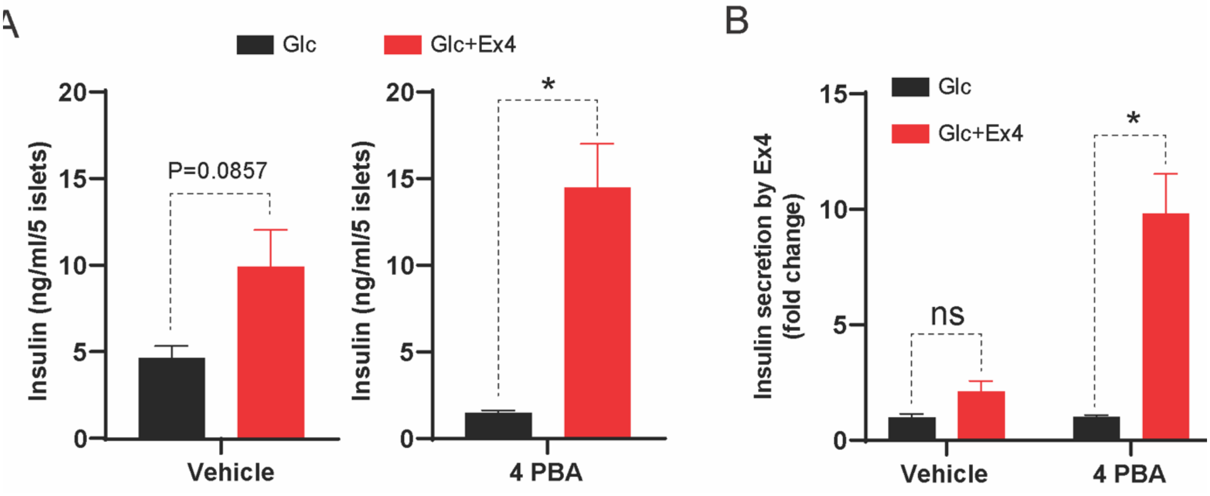

Supplementary Fig. S4. 4-PBA promotes exendin-4-induced insulin secretion in *db/db* islets. (A,B) Islets were isolated from 17-wk-old *db/db* mice and then pretreated with 4-PBA (2.5 mM, 24 h) before high glucose with indicated reagents. (A) Insulin secretion and (B) its fold change. Data are presented as means ± S.E.M (n = 4). Statistical analyses were performed by unpaired t-tests. * $p < 0.05$.

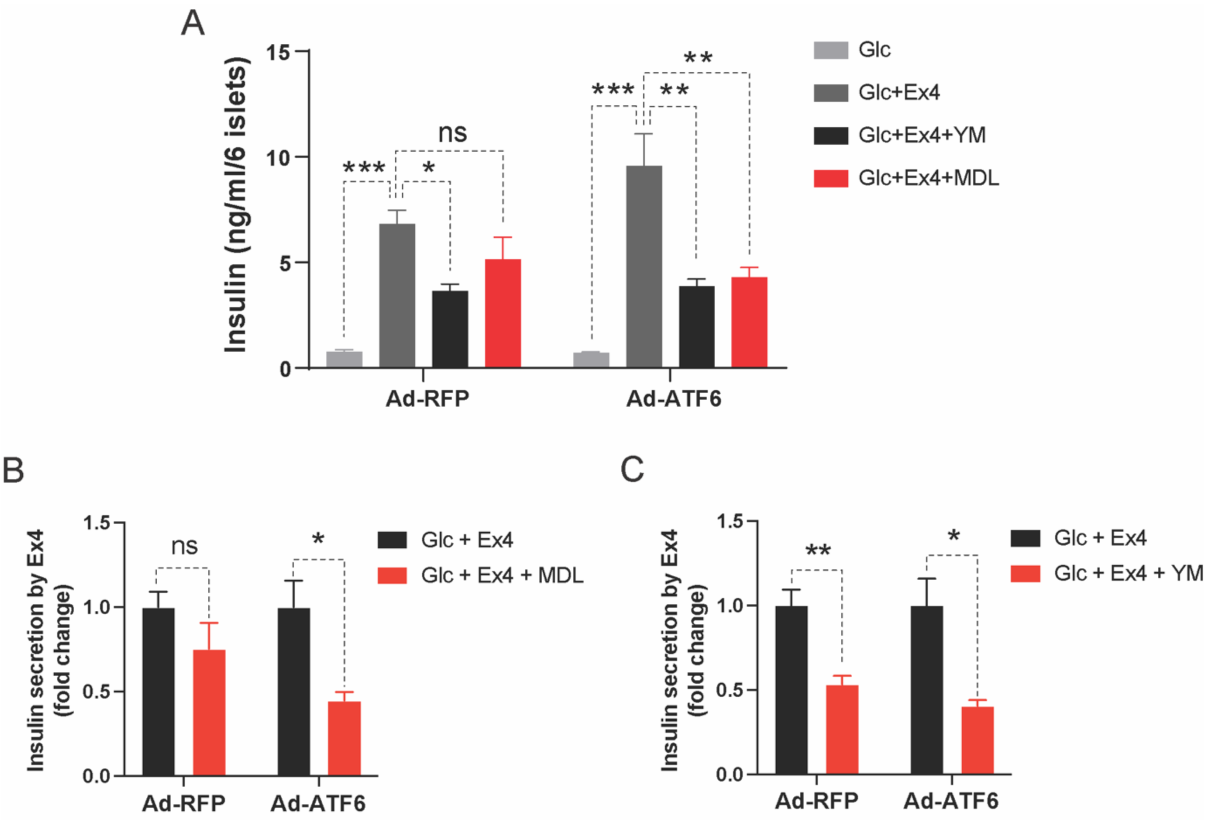

Supplementary Fig. S5. Ectopic expression of ATF6 in ER stress-experiencing islets enhances GLP-1R's signaling utilization of Gs as XBP1. (A–C) Isolated mouse islets were infected with adenovirus expressing RFP or ATF6 and then pretreated with tunicamycin (8 h) before introducing indicated reagents for 1 h. The concentrations of reagents were the same as in previous experiments. (A) Islets' insulin secretion and (B,C) its fold change. Data are presented as means ± S.E.M (n = 4). Statistical analyses were performed by either one-way ANOVA followed (A) or unpaired t-tests (B,C). * $p < 0.05$, ** $p < 0.01$, *** $p < 0.001$.

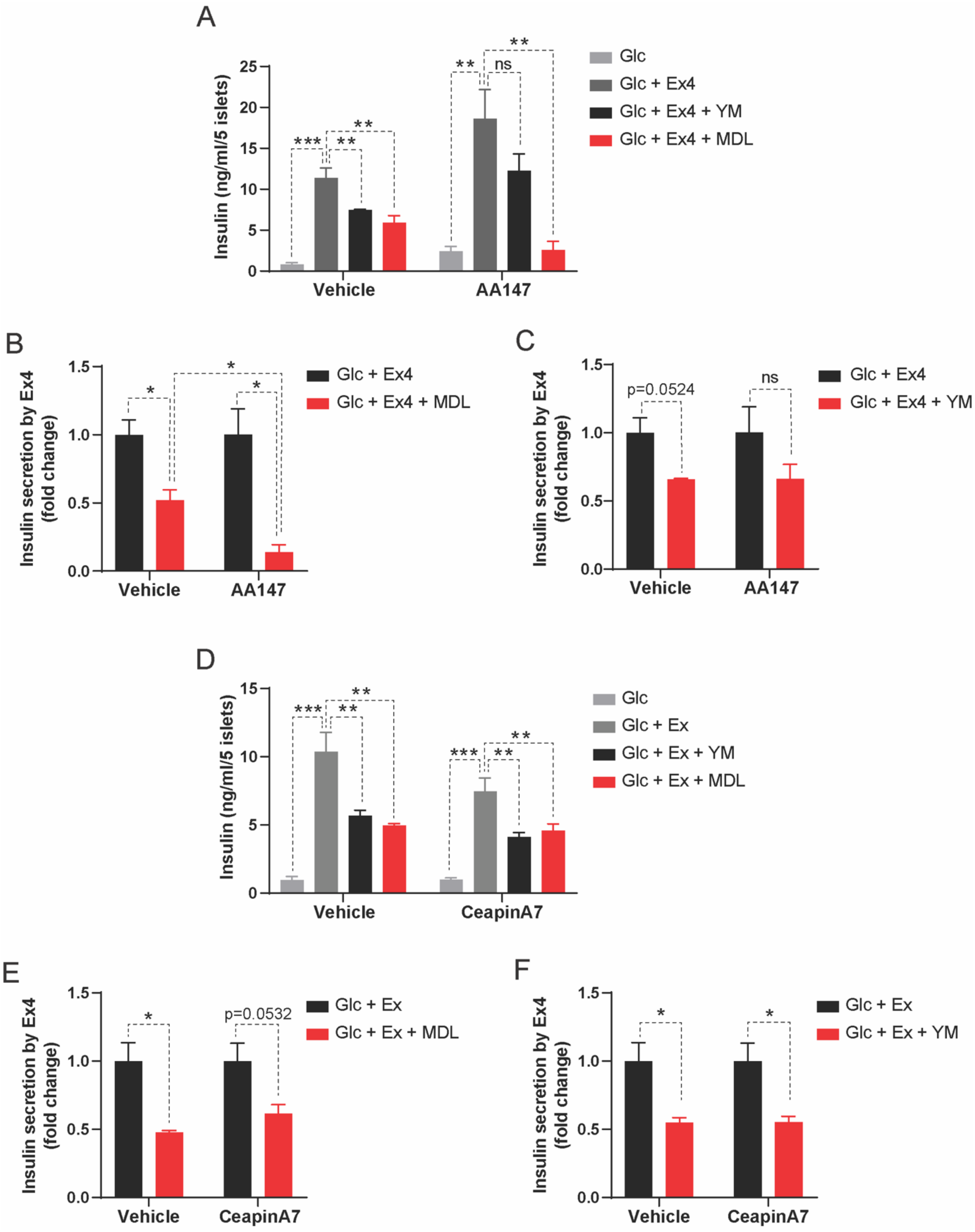

Supplementary Fig. S6. Pharmacological ATF6 activation, rather than inhibition, results in altered GLP-1R's Gs utilization under ER stress. (A–F) Isolated mouse islets were pretreated with ATF6 activator (AA147, 10 μM, 12 h) (A–C) or inhibitor (CeapinA7, 10 μM, 0 h) (D–F) and then tunicamycin (8 h) before introducing indicated reagents for 1 h. The

concentrations of reagents were the same as in previous experiments. (A,D) Islets' insulin secretion and (B,C,E,F) its fold change. Data are presented as means ± S.E.M (n = 4). Statistical analyses were performed by either one-way ANOVA followed (A,D) or unpaired t-tests (B,C,E,F). * $p < 0.05$, ** $p < 0.01$, *** $p < 0.001$.

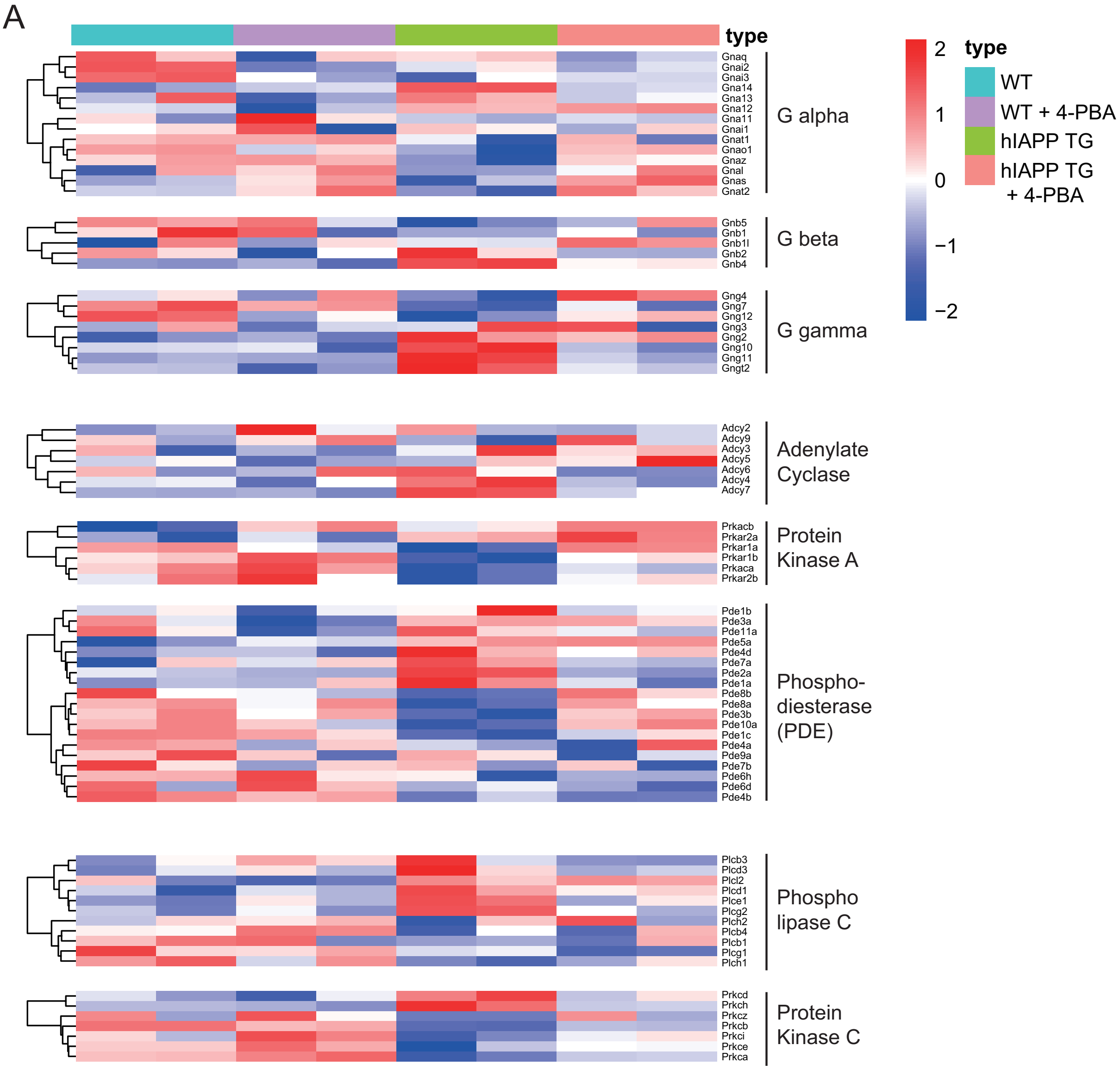

Supplementary Fig. S7. 4-PBA-induced transcriptomic changes of G-protein coupled receptor-related genes. (A) Heatmap analysis of GPCR signaling-related genes from 4-PBA treated islet. The genes are categorized by their molecular function, and their transcriptome data were previously published (GSE84423) [1].

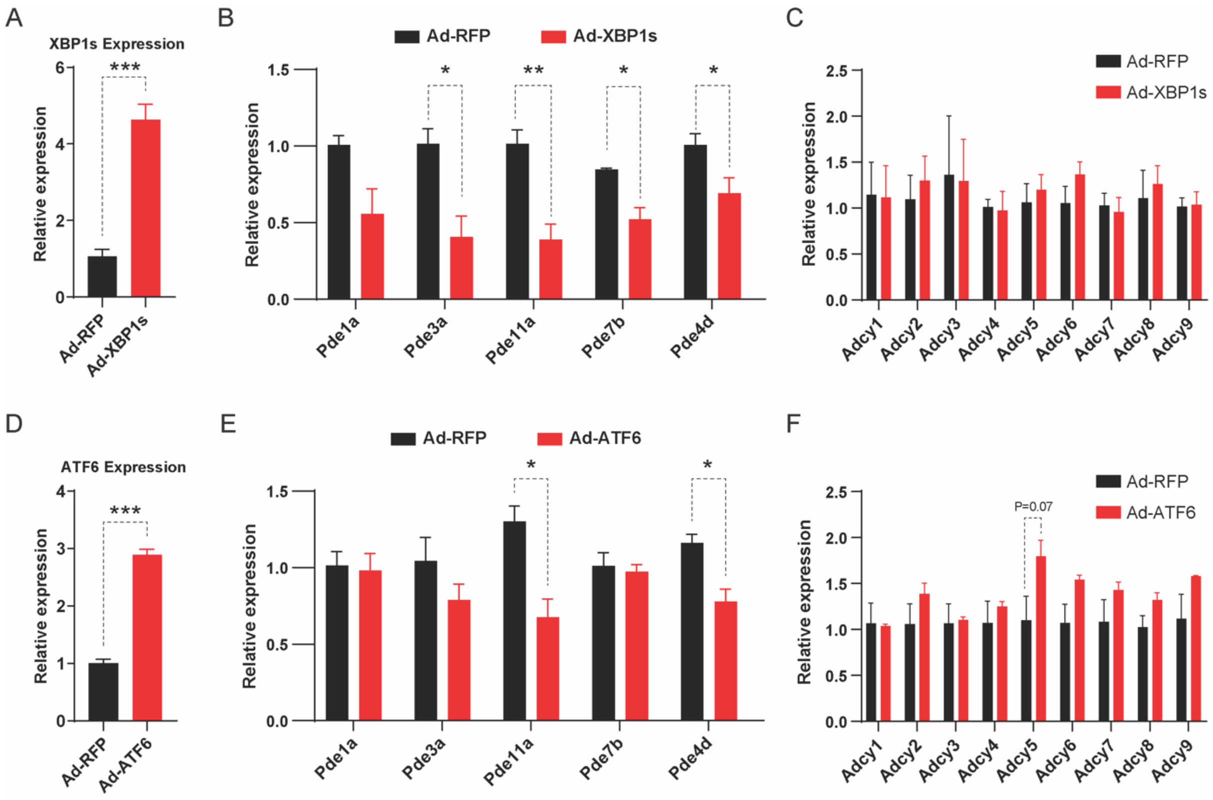

Supplementary Fig. S8. XBP1 and ATF6 modulate cAMP-targeting PDE genes expression. (A–F) The mRNA expression of cAMP-degrading PDEs and adenylyl cyclases (Adcy) in XBP1s- (A–C) or ATF6-expressing islets (D–F). Data are presented as means ± S.E.M (n = 4). Statistical analyses were performed by unpaired t-tests. * $p < 0.05$, ** $p < 0.01$, *** $p < 0.001$.

References

[1] J. Montane, S. de Pablo, C. Castano, J. Rodriguez-Comas, L. Cadavez, M. Obach, M. Visa, G. Alcarraz-Vizan, M. Sanchez-Martinez, A. Nonell-Canals, M. Parrizas, J.M. Servitja, A. Novials, Amyloid-induced beta-cell dysfunction and islet inflammation are ameliorated by 4-phenylbutyrate (PBA) treatment, FASEB J 31 (2017) 5296-5306. 10.1096/fj.201700236R.
